# Supplementary material for: Genomic Characteristics of Stx2e-Producing Escherichia coli Strains Derived from Humans, Animals, and Meats
Source: Pathogens. 2021 Nov 28;10(12):1551. doi: 10.3390/pathogens10121551 (PMC8705337; doi:10.3390/pathogens10121551)
Supplement: Supplementary file 1 [file pathogens-10-01551-s001.zip › pathogens-1436650-supplementary/supplementary_1114/Figure S1.pdf]

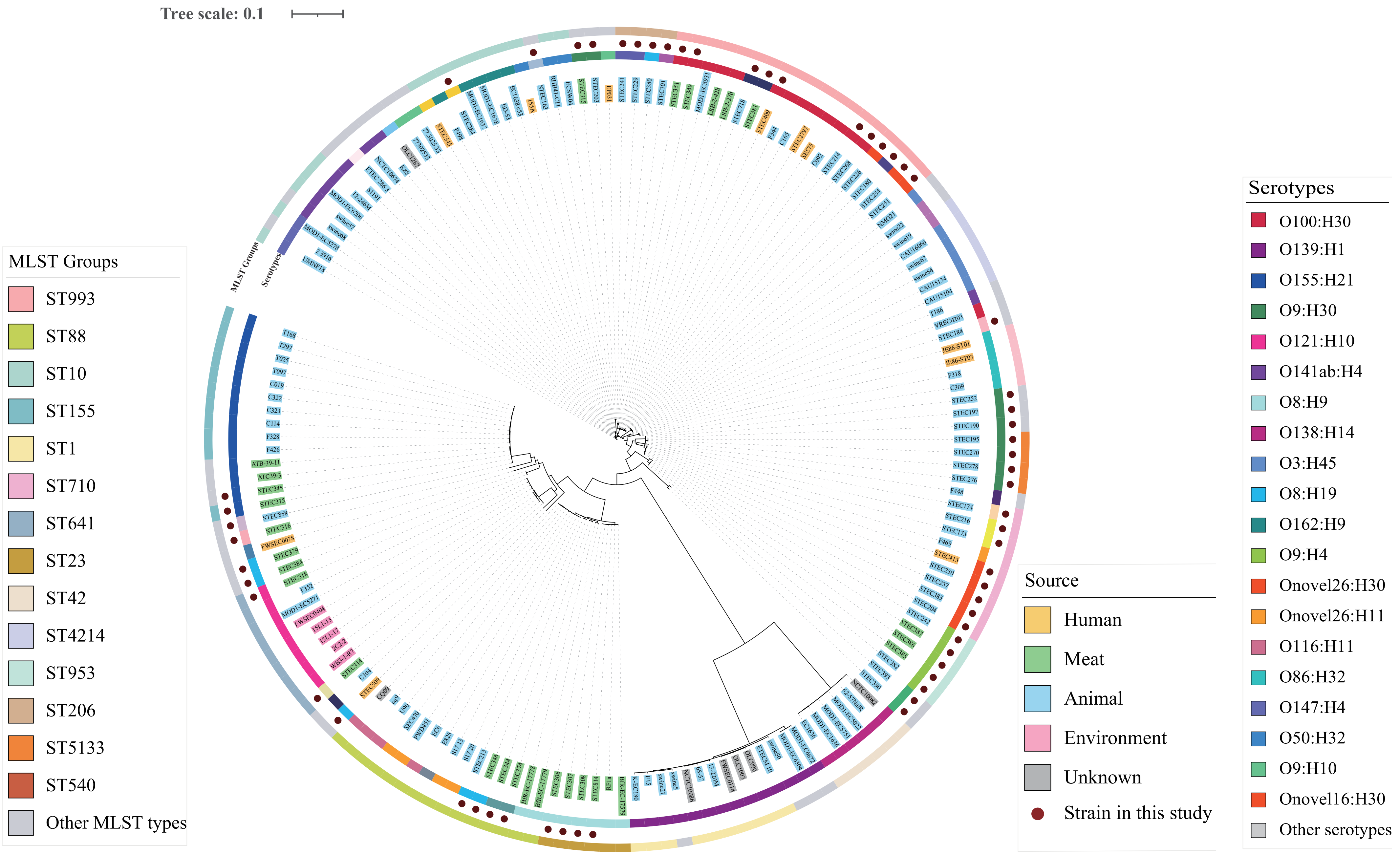

Figure S1. Whole genome phylogeny of Stx2e-STEC strains in this study and reference strains. Stx2e-STEC strains in this study are indicated as shown, others are reference strains used for comparison. The phylogenetic tree was constructed based on core-genome single nucleotide polymorphisms (SNPs) using the Maximum-Likelihood method. The genomes of 102 reference Stx2e- STEC strains were downloaded from NCBI, the accession number, source and country of origin for reference strains are provided in Supplementary Table S1.
