# Supplementary material for: Genomic Characteristics of Stx2e-Producing Escherichia coli Strains Derived from Humans, Animals, and Meats
Source: Pathogens. 2021 Nov 28;10(12):1551. doi: 10.3390/pathogens10121551 (PMC8705337; doi:10.3390/pathogens10121551)
Supplement: Supplementary file 1 [file pathogens-10-01551-s001.zip › pathogens-1436650-supplementary/supplementary_1114/Figure S2.pdf]

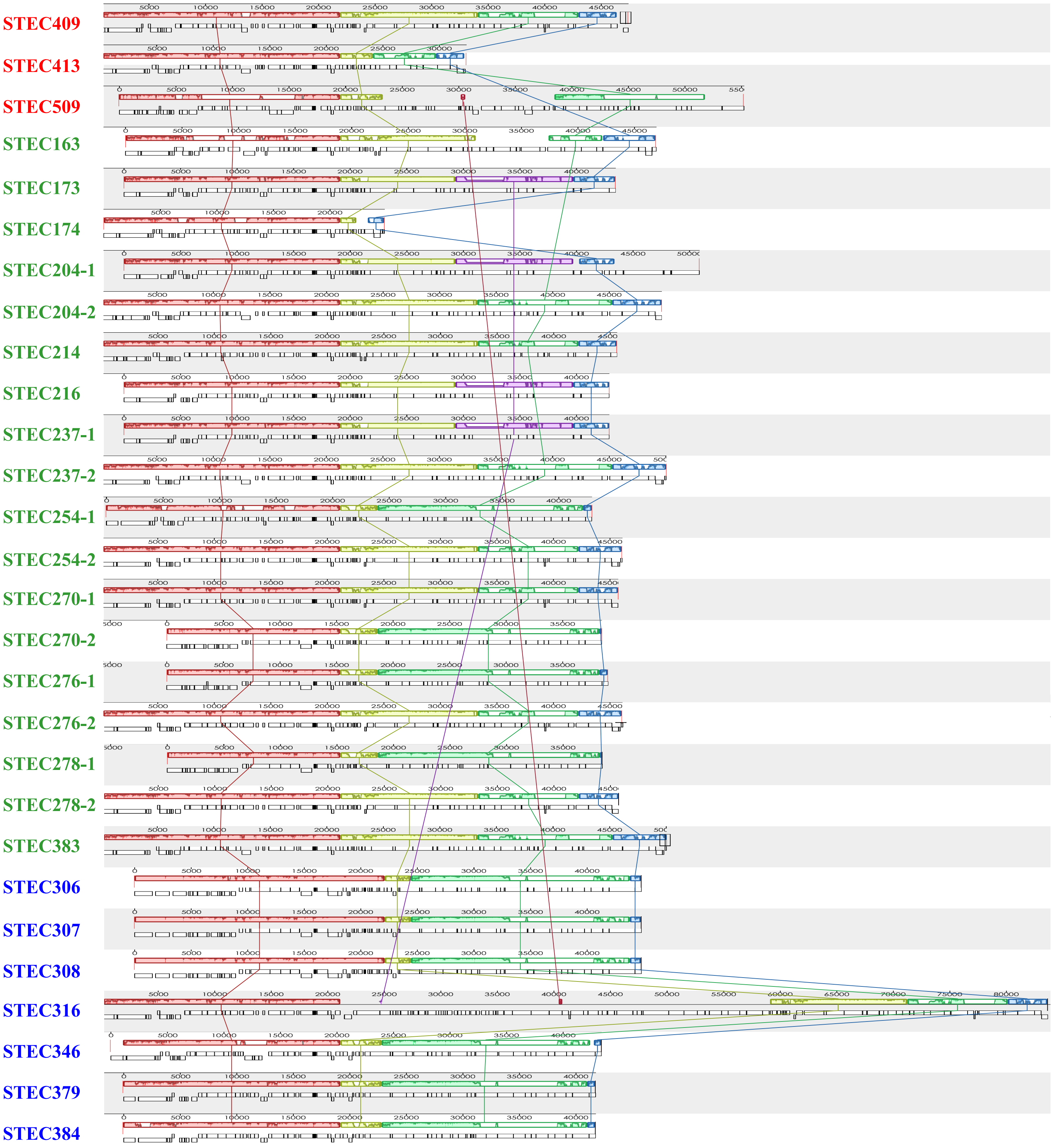


**Figure S2**. Genome alignment of the 28 Stx2e prophages. The color of the text indicates the source of strains, red represents human-derived strains, green represents animal-derived strains and blue represents meat-derived strains. Colored blocks were connected by lines to the homologous in other genomes.
