# Supplementary material for: Genomic Characteristics of Stx2e-Producing Escherichia coli Strains Derived from Humans, Animals, and Meats
Source: Pathogens. 2021 Nov 28;10(12):1551. doi: 10.3390/pathogens10121551 (PMC8705337; doi:10.3390/pathogens10121551)
Supplement: Supplementary file 1 [file pathogens-10-01551-s001.zip › pathogens-1436650-supplementary/supplementary_1114/Supplementary caption.pdf]

Supplementary materials

Table S1. Genomes of 102 Stx2e-STEC strains used in whole genome analysis (.xlsx)

Table S2. Virulence genes in the 59 Stx2e-STEC strains in this study (.xlsx)

Table S3. Characterization of 28 Stx2e-converting prophages (.xlsx)

Figure S1. Whole genome phylogeny of Stx2e-STEC strains in this study and reference strains.

The phylogenetic tree was constructed based on core-genome single nucleotide polymorphisms (SNPs) using the Maximum-Likelihood method. The genomes of 102 reference Stx2e-STEC strains were downloaded from NCBI.

Figure S2. Genome alignment of the 28 Stx2e prophages

The color of the text indicates the source of strains, red represents human-derived strains, green represents animal-derived strains and blue represents meat-derived strains. Colored blocks were connected by lines to the homologous in other genomes.
